# Draft Genome Sequence of Klebsiella pneumoniae UMB7783, Isolated from the Female Bladder

**DOI:** 10.1128/MRA.00394-20

**Published:** 2020-05-07

**Authors:** Erika Balce, Taylor Miller-Ensminger, Adelina Voukadinova, Alan J. Wolfe, Catherine Putonti

**Affiliations:** a Department of Biology, Loyola University Chicago, Chicago, Illinois, USA; b Bioinformatics Program, Loyola University Chicago, Chicago, Illinois, USA; c Department of Microbiology and Immunology, Stritch School of Medicine, Loyola University Chicago, Maywood, Illinois, USA; d Department of Computer Science, Loyola University Chicago, Chicago, Illinois, USA; Georgia Institute of Technology

## Abstract

Klebsiella pneumoniae is a Gram-negative bacterium that is highly pathogenic and frequently resistant to antibiotics. Here, we report the draft genome sequence of K. pneumoniae strain UMB7783, isolated from the female bladder.

## ANNOUNCEMENT

Klebsiella pneumoniae is an opportunistic pathogen that is typically harmless to healthy hosts but dangerous to those who are immunocompromised (1). In a survey of 183 hospitals across the United States, *Klebsiella* species were found to be the third most common cause of hospital-acquired infections (2). Furthermore, urinary tract infections (UTIs), which account for an estimated 6 to 17% of *Klebsiella* infections, are most frequently infections caused by K. pneumoniae (3). K. pneumoniae is difficult to treat due to its high resistance to many antibiotics, including beta-lactams (1, 4). Here, we present the genome of a single K. pneumoniae strain isolated from a catheterized urine sample obtained from a woman with a recurrent urinary tract infection (rUTI).

K. pneumoniae UMB7783 was collected as part of a prior institutional review board (IRB)-approved study (University of California, San Diego, IRB no. 170077AW) using the expanded quantitative urine culture (EQUC) method (5). The genus and species for this isolate were determined using matrix-assisted laser desorption ionization–time of flight (MALDI-TOF) mass spectrometry. The isolate was then stored at –80°C until sequencing. The K. pneumoniae isolate was first streaked onto a Columbia nalidixic acid (CNA) agar plate and incubated at 35°C with 5% CO_2_ for 24 h. A single colony was selected from the plate, placed in brain heart infusion (BHI) medium, and incubated at 35°C with 5% CO_2_ for 24 h. DNA was extracted with the Qiagen DNeasy blood and tissue kit using the manufacturer’s Gram-positive extraction protocol with some minor exceptions. Rather than the recommended 180 μl of lysis buffer, 230 μl was added (180 μl of 20 mM Tris-Cl, 2 mM sodium EDTA, and 1.2% Triton X-100 and 50 μl of lysozyme). Additionally, the sample was incubated at 56°C for 10 min in step 5 of the protocol. The DNA was quantified using the Qubit fluorometer and sent to the Microbial Genomic Sequencing Center (MiGS) at the University of Pittsburgh for library preparation and sequencing. The DNA was first enzymatically fragmented using an Illumina tagmentation enzyme. Indices were attached using PCR and sequenced using an Illumina NextSeq 550 flow cell, producing 1,384,660 pairs of 150-bp reads. Raw reads were trimmed using Sickle v1.33 (https://github.com/najoshi/sickle) and assembled using SPAdes v3.13.0 with the “only-assembler” option for k values of 55, 77, 99, and 127 (6). Genome annotation was performed using PATRIC (7). The NCBI Prokaryotic Genome Annotation Pipeline (PGAP) v4.11 (8) also was used to annotate the genome assembly, and the annotation is publicly available. The genome assembly was also screened for prophages using the Web tool PHASTER (9) and for antibiotic resistance genes using the Center for Genome Epidemiology’s Web tool ResFinder v3.2 (10). Unless noted, default parameters were used for each software tool.

The K. pneumoniae UMB7783 draft genome is 5,667,345 bp long, assembled in 154 contigs, with a GC content of 57.19%, a genome coverage of 62×, and an *N*_50_ score of 119,241 bp. PGAP identified 5,744 genes total, including 5,522 protein coding genes, 4 rRNA operons, and 80 tRNAs. PHASTER found 4 intact phages for this strain on 4 different contigs. Two of these predicted intact phages (30.2 kbp and 27.1 kbp long) are integrated; they are encoded within significantly larger contigs. The other 2 (22.9 kbp and 17.2 kbp long) were found on independent contigs in which the predicted phage’s sequence was essentially the entire contig sequence. Additionally, the PATRIC annotation predicted that the strain had both piperacillin-tazobactam and tetracycline resistance. Further, ResFinder identified SHV, which is associated with beta-lactam resistance. This is a well-known antibiotic resistance determinant for the species (4). ResFinder also identified genes conferring resistance to fosfomycin (*fosA*) and quinolone (*oqxA* and *oqxB*).

Further analysis of K. pneumoniae genomes from individuals with rUTIs, such as the female patient from which this strain was isolated, will improve our understanding of this highly pathogenic organism within the urinary tract. Furthermore, genomic studies of such isolates will inform the development of treatments, given high levels of antibiotic resistance.

### Data availability.

This whole-genome shotgun project has been deposited in GenBank under accession no. JAAUVW00000000. The version described in this paper is the first version, JAAUVW01000000. The raw sequencing reads have been deposited in the SRA under accession no. SRR11441027.
